# A comparison between seven scales of neuropsychological assessments for cognitive impairment screening in Chinese older population: a cross-sectional study in Chongqing, China

**DOI:** 10.3389/fpsyt.2026.1786091

**Published:** 2026-07-23

**Authors:** Feiya Cai, Xiaolin Tan, Bo Tian

**Affiliations:** Department of Psychiatric Research, Chongqing Mental Health Center, Chongqing, China

**Keywords:** ADL, agreement, CDR, correlation, cognitive impairment, Mini-Cog, risk factors

## Abstract

**Background:**

The Clinical Dementia Rating Scale (CDR), the Ascertain Dementia 8 (AD8), the Mini-Cog, the Verbal Fluency Test (VFT), the Community Screener for Dementia (CSI-D), the Rey Auditory Verbal Learning Test (RAVLT), and the Activities of Daily Living Scale (ADL) represent seven commonly employed methods for community cognitive impairment screening in China that have garnered limited attention. This study aimed to assess the discriminatory power of these seven tests when administered concurrently in a single screening to detect cognitive impairment in the absence of a gold standard.

**Methods:**

We conducted a cross-sectional survey among 1,506 elderly people aged 60 and above in the community. The cognitive and social functions of the elderly population were evaluated by using the Seven scales. The characteristics related to demographics and health were collected through questionnaire surveys, and the correlations and consistencies of the Seven scales with cognitive impairment were analyzed respectively. Multifactor logistic regression model was used to analyze the risk factors of cognitive impairment in each scale.

**Results:**

The positive screening proportions for the seven scales—CDR, ADL, AD8, CSI-D, Mini-Cog, VFT, and RAVLT—were 61.1%, 33.7%, 38.0%, 37.1%, 53.7%, 39.3%, and 52.9%, respectively. Agreement on cognitive impairment risk between each pair of scales was fair-to-moderate (Kappa: 0.32–0.645, all *p* < 0.001). Screening results differed significantly across the seven scales by age, educational, marital status, epilepsy, cerebral infarction, brain atrophy, and depression (all P < 0.001). Notably, Screen-positive proportions for cognitive impairment rose significantly with increasing age (p < 0.001); the ≥ 80-year-old group showed the highest proportions across scales. Conversely, higher educational attainment was associated with lower risk of screening positive for cognitive impairment (p < 0.001).

**Conclusions:**

Our study identified a relatively high screening positivity proportion for cognitive impairment in Chongqing, Age, Sex, Education, Marital status, Brain atrophy, Anxiety, and Depression are risk factors for screening positivity. The identified screening positivity for cognitive impairment varied across different neuropsychological assessment methods, indicating that assessment choice should be tailored to population characteristics rather than applying a one-size-fits-all approach. Selecting the appropriate tool can improve sensitivity and reduce missed diagnoses.

## 1 Introduction

Dementia denotes cognitive impairment arising from diverse brain diseases and injuries (1). According to WHO reports in 2021, 57 million people worldwide were living with dementia, more than 60% of whom resided in low- and middle-income countries, and nearly 10 million new cases occurred annually; dementia ranked as the seventh leading cause of death (2). By the end of 2024, China’s population aged 60 years and older reached 310 million, representing 22.0% of the total population. As a large developing country, China therefore faces a growing dementia burden that parallels the peak of population aging (3). One report showed that in 2021 China’s age-standardized dementia prevalence reached 900.8 per 100,000—the highest among 204 countries—underscoring the escalating public health challenge (4). Because most dementia begin insidiously, progress irreversibly, and currently lack effective cures, research has shifted toward delaying progression at the preclinical stage, especially among people with cognitive impairment (CI) (5). CI signifies reduced cognitive performance without implying a cognitive disorder or clinical diagnosis, yet the potential for Mild cognitive impairment(MCI) and dementia remains. MCI denotes a progressive decline in memory or other cognitive domains that does not substantially impair activities of daily living and does not meet diagnostic criteria for dementia; it therefore represents a transitional stage between normal cognitive aging and dementia (6). In China, MCI prevalence among older adults is high: epidemiological surveys estimate that approximately 38.77 million people aged 60 and above have MCI, corresponding to an overall prevalence of 15.5% (7). Within 3 to 5 years: roughly one-third of MCI patients remain stable, one-third revert to normal cognition, and one-third progress to dementia (8). Thus, MCI is considered a “golden window” for interventions aimed at delaying dementia onset and progression, making large-scale community screening and the identification of high-risk factors in older adults essential for preventing conversion (9). Previous studies indicate that the incidence of recovery from mild cognitive impairment (MCI) to normal cognition is 31% (1). Consequently, identifying the controllable factors associated with CI in Chongqing can offer a theoretical foundation for clinicians in the management and treatment of this condition.

Neuropsychological assessment is essential for diagnosing and studying cognitive impairment (CI) because it evaluates cognitive function and can detect CI with brief tests (10). The Mini-Mental State Examination (MMSE) is heavily affected by educational level and lacks sensitivity for differentiating CI from Alzheimer’s disease (AD) or from cognitively normal individuals; thus, it has limited value for CI detection and is unsuitable for community screening (11, 12). The Montreal Cognitive Assessment (MoCA) assesses a wider range of cognitive domains than the MMSE but shows a relatively high false-positive rate, and the evidence that it reliably separates normal older adults from those with CI is only level II (13); therefore, it is not recommended for community screening. Prior work has focused mainly on these two instruments and has insufficiently examined alternative scales; consequently, this study does not recompare the MMSE and MoCA.

This study targets practical community screening and therefore includes seven neuropsychological instruments commonly used for CI detection in Chinese communities: the Clinical Dementia Rating (CDR) Scale (14), Ascertain Dementia 8 (AD8) (15), Mini-Cog, Verbal Fluency Test (VFT) (16), Community Screening Instrument for Dementia (CSI-D) (17), Rey Auditory Verbal Learning Test (RAVLT) (18), and the Activity of Daily Living Scale (ADL) (19). Their combined use balances comprehensiveness with simplicity and field practicality. Prior work has been limited by single-center or region-specific designs, small samples, and comparisons restricted to two or three measures; these limitations reduce representativeness, reliability, and the scope of clinical conclusions (14, 20). To address these gaps, we conducted a multicenter study to assess concordance among these seven instruments in identifying CI, Because this study has a larger and more representative sample, it may yield different or novel findings.

Understanding the screening positivity proportion for CI in Chongqing and identifying potentially modifiable risk factors are essential for informing prevention, treatment, and intervention strategies, and may suggest new ways to delay dementia onset and progression. Prior studies indicate that sociodemographic variables (21, 22), physiological and biochemical marker (4, 23), and medical history have predictive value for CI and dementia risk (24, 25); examples include age, sex, education, marital status, and chronic disease history (22, 26). However, findings are partly inconsistent, which likely reflects differences in sampling countries, lifestyle and environmental contexts, research methodologies, age ranges, neuropsychological assessment methods, and diagnostic criteria—each of which can affect outcomes (27, 28). Adopting a pluralistic and open approach to research, we selected sociodemographic and health-related variables for this study by synthesizing prior findings (27, 29) and considering Chongqing’s local characteristics (30, 31). This strategy aligns with available scientific evidence while capturing features specific to Chongqing’s elderly population. Finally, by comparing our results with studies from other countries and other Chinese provinces or municipalities, we aim to identify CI screening and intervention strategies adapted to local conditions.

In summary, this study aims to quantify the correlations and concordance among CDR, AD8, VFT, CSI-D, RAVLT, ADL, and Mini-Cog in detecting CI and to examine how each test differentially associates with factors that influence screening outcomes for CI. The results could substantially inform the choice of neuropsychological instruments for community-based CI screening and guide prevention and management strategies in community settings.

## 2 Methods

### 2.1 Study population

**Figure 1:**
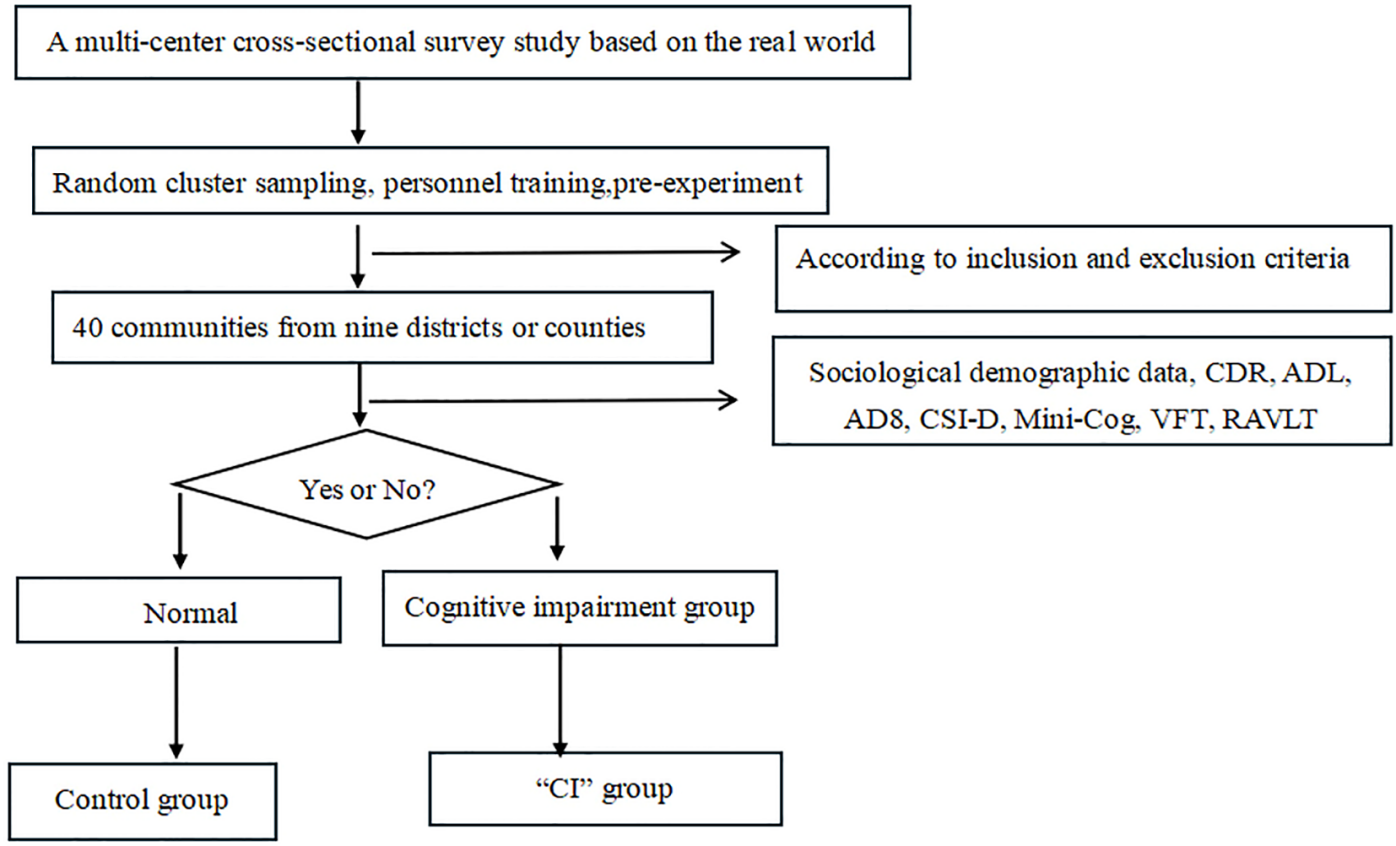
Flowchart. A cross-sectional investigation study based on seven scales of neuropsychological assessments.

This descriptive, observational, cross-sectional study was conducted in Chongqing City from January 2024 to November 2024. Using stratified cluster sampling, we randomly selected 40 communities across 9 districts and counties to collect sociodemographic data, medical histories, and neuropsychological assessment scores from older adults. Eligible participants met all of the following criteria (1): Age ≥ 60 years, (2) Permanent residence in the sampled community, (3) Clear consciousness with the ability to communicate, (4) Comprehension of Chongqing dialect or Mandarin, and (5) No diagnosis of Alzheimer’s disease or other dementias. Exclusion criteria comprised seizure phase of epilepsy, recent cerebral hemorrhage or infarction, congenital or acquired intellectual disability, severe visual or hearing impairment, severe anxiety or depression, diagnosed CI, and inability to perform activities of daily living. The detailed screening procedure is shown in **Figure 1**.

### 2.2 General questionnaire

The investigators developed a Case Report Form (CRF) to collect relevant data for the study. The CRF records participants’ sociodemographic information and medical history, including age, sex, education level, marital status, smoking, alcohol use, hypertension, diabetes, hyperlipidemia, heart disease, cerebrovascular disease, Parkinson’s disease, epilepsy, anxiety, depression, and insomnia. The disease history in this study—including definitions of hypertension, diabetes, brain atrophy, depression, anxiety, epilepsy, insomnia, and other conditions—was obtained solely from patient self-reports. No standardized clinical examinations were performed to confirm these diagnoses.

### 2.3 Cognitive assessment

Neuropsychological assessment of CI needs to comprehensively consider cognitive and non-cognitive domains. Because assessment instruments vary in design and scoring, clinicians combine them flexibly to address specific needs. The specific definitions and scoring criteria for the seven neuropsychological assessment scales are listed in **Table 1**. Note that the outcome “CI” in this study denotes an individual’s current cognitive decline as assessed by the applied scales and their established cutoffs. CI reflects only the scales’ assessment results and should not be interpreted as a definitive or clinical diagnosis.

**Table 1:** The information on seven cognitive assessment scales.

Scales	Project content	Classification of dysfunction
CDR	Assess six core areas: memory, orientation, judgment and problem solving, community affairs, family and hobbies, and personal self-care.	Classify functioning into five levels: 0: no dementia; 0.5: suspected dementia; 1: mild dementia, defined by marked memory impairment plus mild impairment in at least one other area; 2: moderate dementia; 3: Severe dementia.
ADL	The scale comprises 14 items grouped into two categories. The first category covers physical self-care (6 items): feeding, dressing, washing/toileting hygiene, toileting, ambulation, and stair climbing. The second category covers instrumental activities (8 items): telephone use, shopping, cooking, housekeeping, laundry, using transportation, medication management, and financial management.	A total score range of 14–56 points. Scores ≤16 indicate normal function; 17–22 indicate mild impairment; 23–28 indicate moderate impairment; and scores ≥29 indicate severe impairment.
AD8	Eight items: memory decline, reduced capacity for handling complex tasks, language impairment, visuospatial decline, decreased judgment, changes in interests, decline in activities of daily living, and emotional or behavioral changes.	A total score from 0 to 8. A score ≥2 indicates possible cognitive impairment, <2 suggests a high likelihood of normal cognitive function.
CSI-D	Consisting of two components: cognitive assessment scores and informant-rated scores	Cognitive assessment: Scores ≤4 indicate severe dementia, scores of 5–6 require an informant-rated assessment, and scores ≥7 make dementia highly unlikely. Informant-rated scores: ≤1 indicate normal cognition, while scores ≥2 indicate dementia.
Mini-cog	The Mini-Cog consists of a three-item immediate recall task paired with the Clock Drawing Test (CDT). Participants first hear three unrelated words (for example: apple, watch, flag), then complete the CDT, and finally attempt to recall the three words.	After the clock-drawing test, 1 point is given for each correctly recalled word (0–3 points). Correct placement of the clock face is scored as 2 points; any error yields 0 points. The Mini-Cog score equals the sum of the noun-recall and clock-drawing scores. A total of 0–2 indicates a positive screen for dementia.
VFT	Task: Name as many words as possible from a specified category (e.g., animals, fruits, vegetables)	Within 1 minute. Scoring: ≥10 items in 1 minute = normal; 5–9 items = mild impairment; <5 items = moderate to severe impairment.
RAVLT	The assessment consisted of two memory-test phases: immediate recall and delayed recall.	Each phase had a maximum of 15 points, with one point awarded for each correctly recalled word. The two phase scores were summed to produce the final score.

CDR, the clinical dementia rating scale. ADL, the activity of daily living scale. AD8, ascertain dementia 8. CSI-D, community screening instrument for dementia. Mini-cog, Mini-cognitive Assessment. VFT, the verbal fluency test. RAVLT, the rey auditory verbal learning test.

### 2.4 Data collection

All research team members completed standardized training in the administration of neuropsychological assessment instruments and passed competency evaluations before the study began. At the start of the formal investigation, researchers explained the study’s purpose, significance, participant rights and obligations, and precautions, and they informed participants that withdrawal at any time would have no adverse consequences; written informed consent was obtained before data collection. Each questionnaire was independently reviewed by two psychiatrists at the attending level or above; any identified issues were clarified with the participant and the questionnaire was corrected accordingly. If a potential participant already exhibited overt cognitive symptoms and thus failed to meet inclusion criteria, researchers assisted in contacting family members to arrange further diagnostic and treatment services, providing humane care while upholding the principle of beneficence.

### 2.5 Ethics statement

The study adhered to the Declaration of Helsinki and its amendments, approved by the ethics committee of Chongqing Mental Health Center (Ethical Medical Case No. (022) of 2023). All participants have written informed consent before recruitment and their personal information and survey data would be kept strictly confidential.

### 2.6 Statistical analysis

Data integrity and logical consistency were checked in Microsoft Excel. R Studio 4.4.3 was used for correlation analyses, chi-square tests, and construction of logistic regression models. Categorical variables are reported as n (%). Chi-square tests compared CI-screening positivity distributions across scales by relevant factors. Cramer’s V assessed correlations among the scales, and the results were displayed in a matrix heatmap. Cohen’s kappa coefficient evaluated agreement between scales. Multivariable logistic regression examined associations between sociodemographic and health-related factors and CI-screening positivity as determined by each scale. Variables that showed statistically significant differences in CI-screening positivity distribution in this study, together with sex—which prior studies identify as a strong determinant of CI—were entered simultaneously into each model. No multicollinearity was detected in the seven final models (Tolerance: 0.801–0.972 and VIF: 1.029–1.249 for ADL; Tolerance: 0.715–0.971 and VIF: 1.03–1.4 for CDR; Tolerance: 0.715–0.97 and VIF: 1.031–1.398 for AD8; Tolerance: 0.723–0.972 and VIF: 1.029–1.383 for CSI-D; Tolerance: 0.807–0.981 and VIF: 1.02–1.24 for Mini-cog; Tolerance: 0.833–0.973 and VIF: 1.02–1.201 for VFT; Tolerance: 0.809–0.973 and VIF: 1.028–1.236 for RAVLT). *P* < 0.05 were considered statistically significant (**Supplementary Table 1**).

## 3 Results

### 3.1 Characteristics of study population

The study enrolled 1,506 individuals aged 60 and older. Participants aged 60–69, 70–79, and ≥80 accounted for 28.2%, 41.3%, and 30.5% of the sample, respectively. Women comprised 73% of the cohort and men 27%. Educational attainment was distributed as follows: illiterate 16.9%, primary school 34.3%, and junior high school or higher 48.8%. Marital status showed 58.6% were married and 41.4% unmarried. Prevalence of comorbid conditions was: hypertension 49.7%, hyperglycemia 23.8%, hyperlipidemia 22.1%, coronary heart disease 22.1%, Parkinson’s disease 5.4%, epilepsy 1.0%, cerebral infarction 25.2%, cerebral atrophy 38.6%, cerebral hemorrhage 2.9%, and brain trauma 8.7%. Smoking and alcohol use were reported by 8.6% and 11.8% of participants, respectively. Reported rates of depression, anxiety, and insomnia were 1.1%, 1.1%, and 10.0%, respectively (**Table 2**). All seven screening scales showed significant differences in screening positivity for CI across age groups, education levels, marital status, epilepsy, cerebral infarction, brain atrophy, and depression (p<0.05). Other variables reached significance on some scales, including gender, hypertension, hyperglycemia, hyperlipidemia, coronary heart disease, Parkinson’s disease, cerebral hemorrhage, brain trauma, smoking, alcohol use, anxiety, and insomnia(**Table 2**). Multiple comparisons indicated that participants aged 60–69, those with at least a primary school education, married individuals, and participants without histories of epilepsy, cerebral infarction, brain atrophy, or depression were more likely to score in the lower-risk range on all seven screening scales (all *p* < 0.05).

**Table 2:** A comparison of the differences in the risk distribution of screening-positive for CI among sociodemographic and health-related factors in the study population for the seven scales.

Characteristics	N(%)	CDR		ADL		AD8		CSI-D	
		n(%)	*p*-value	n(%)	*p*-value	n(%)	*p*-value	n(%)	*p*-value
Age(years)			<0.001		<0.001		<0.001		<0.001
60-69	425(28.2)	196(46.1)		67(15.8)		92(21.6)		93(21.9)	
70-79	622(41.3)	358(57.6)		162(26)		201(32.3)		215(34.6)	
80-	459(30.5)	366(79.7)		278(60.6)		280(61)		251(54.7)	
Gender			0.122		0.033		0.513		0.066
male	406(27.0)	261(64.3)		154(37.9)		149(36.7)		166(40.9)	
female	1100(73.0)	659(59.9)		353(32.1)		424(38.5)		393(35.7)	
Educational attainment			<0.001		0.005		<0.001		<0.001
Illiteracy	255(16.9)	198(77.6)		100(39.2)		136(53.3)		123(48.2)	
primary school	516(34.3)	328(63.6)		147(28.5)		173(33.5)		185(35.9)	
junior high school or above	735(48.8)	394(53.6)		260(35.4)		264(35.9)		251(34.1)	
Marital status			<0.001		<0.001		<0.001		<0.001
no	623(41.4)	429(68.9)		274(44)		291(46.7)		278(44.6)	
yes	883(58.6)	491(55.6)		233(26.4)		282(31.9)		281(31.8)	
Hypertension			0.01		0.001		0.026		0.004
no	757(50.3)	438(57.9)		225(29.7)		267(35.3)		254(33.6)	
yes	749(49.7)	482(64.4)		282(37.7)		306(40.9)		305(40.7)	
Hyperglycemia			0.057		0.009		0.331		0.164
no	1148(76.2)	686(59.8)		366(31.9)		429(37.4)		415(36.1)	
yes	358(23.8)	234(65.4)		141(39.4)		144(40.2)		144(40.2)	
Hyperlipidemia			0.56		0.006		0.014		0.143
no	1173(77.9)	712(60.7)		374(31.9)		427(36.4)		424(36.1)	
yes	333(22.1)	208(62.5)		133(39.9)		146(43.8)		135(40.5)	
Coronary heart disease			0.013		0.001		0.037		0.111
no	1173(77.9)	697(59.4)		369(31.5)		430(36.7)		423(36.1)	
yes	333(22.1)	223(67)		138(41.4)		143(42.9)		136(40.8)	
Parkinson's Disease			0.001		<0.001		0.002		0.243
no	1425(94.6)	856(60)		465(32.6)		529(37.1)		524(36.8)	
yes	81(5.4)	64(79)		42(51.9)		44(54.3)		35(43.2)	
Epilepsy			0.01		<0.001		0.001		0.001
no	1491(99.0)	906(60.8)		495(33.2)		561(37.6)		547(36.7)	
yes	15(1.0)	14(93.3)		12(80)		12(80)		12(80)	
Cerebral infarction			<0.001		<0.001		<0.001		<0.001
no	1127(74.8)	626(55.5)		299(26.5)		357(31.7)		350(31.1)	
yes	379(25.2)	294(77.6)		208(54.9)		216(57)		209(55.1)	
Cerebral atrophy			<0.001		<0.001		<0.001		<0.001
no	925(61.4)	505(54.6)		266(28.8)		294(31.8)		301(32.5)	
yes	581(38.6)	415(71.4)		241(41.5)		279(48)		258(44.4)	
Cerebral Hemorrhage			0.002		<0.001		0.001		<0.001
no	1463(97.1)	884(60.4)		479(32.7)		546(37.3)		532(36.4)	
yes	43(2.9)	36(83.7)		28(65.1)		27(62.8)		27(62.8)	
Brain trauma			0.704		0.078		0.685		0.287
no	1375(91.3)	842(61.2)		472(34.3)		521(37.9)		516(37.5)	
yes	131(8.7)	78(59.5)		35(26.7)		52(39.7)		43(32.8)	
Smoking			0.912		0.001		0.002		0.079
no	1376(91.4)	840(61)		480(34.9)		540(39.2)		520(37.8)	
yes	130(8.6)	80(61.5)		27(20.8)		33(25.4)		39(30)	
Drinking			<0.001		<0.001		<0.001		0.003
no	1328(88.2)	834(62.8)		471(35.5)		531(40)		511(38.5)	
yes	178(11.8)	86(48.3)		36(20.2)		42(23.6)		48(27)	
Depression			<0.001		<0.001		0.005		0.001
no	1489(98.9)	903(60.6)		492(33)		561(37.7)		546(36.7)	
yes	17(1.1)	17(100)		15(88.2)		12(70.6)		13(76.5)	
Anxiety			0.191		0.006		0.023		0.174
no	1489(98.9)	907(60.9)		496(33.3)		562(37.7)		550(36.9)	
yes	17(1.1)	13(76.5)		11(64.7)		11(64.7)		9(52.9)	
Insomnia			0.003		<0.001		0.01		0.008
no	1355(90.0)	811(59.9)		435(32.1)		501(37)		488(36)	
yes	151(10.0)	109(72.2)		72(47.7)		72(47.7)		71(47)	
Characteristics	N(%)	Mini-cog		VFT		RAVLT			
		n(%)	*p*-value	n(%)	*p*-value	n(%)	*p*-value		
Age(years)			<0.001		<0.001		<0.001		
60-69	425(28.2)	163(38.4)		112(26.4)		164(38.6)			
70-79	622(41.3)	308(49.5)		219(35.2)		305(49)			
80-	459(30.5)	338(73.6)		261(56.9)		328(71.5)			
Gender			0.249		0.001		0.195		
male	406(27.0)	228(56.2)		188(46.3)		226(55.7)			
female	1100(73.0)	581(52.8)		404(36.7)		571(51.9)			
Educational attainment			<0.001		<0.001		<0.001		
Illiteracy	255(16.9)	183(71.8)		125(49)		178(69.8)			
primary school	516(34.3)	288(55.8)		208(40.3)		280(54.3)			
junior high school or above	735(48.8)	338(46)		259(35.2)		339(46.1)			
Marital status			<0.001		0.002		<0.001		
No	623(41.4)	373(59.9)		274(44)		370(59.4)			
Yes	883(58.6)	436(49.4)		318(36)		427(48.4)			
Hypertension			0.13		0.01		0.006		
No	757(50.3)	392(51.8)		273(36.1)		374(49.4)			
Yes	749(49.7)	417(55.7)		319(42.6)		423(56.5)			
Hyperglycemia			0.075		0.25		0.582		
No	1148(76.2)	602(52.4)		442(38.5)		603(52.5)			
Yes	358(23.8)	207(57.8)		150(41.9)		194(54.2)			
Hyperlipidemia			0.912		0.438		0.087		
No	1173(77.9)	631(53.8)		455(38.8)		607(51.7)			
Yes	333(22.1)	178(53.5)		137(41.1)		190(57.1)			
Coronary heart disease			0.045		0.199		0.18		
No	1173(77.9)	614(52.3)		451(38.4)		610(52)			
Yes	333(22.1)	195(58.6)		141(42.3)		187(56.2)			
Parkinson's Disease			0.086		0.002		0.001		
No	1425(94.6)	758(53.2)		547(38.4)		739(51.9)			
Yes	81(5.4)	51(63)		45(55.6)		58(71.6)			
Epilepsy			0.01		<0.001		0.035		
No	1491(99.0)	796(53.4)		579(38.8)		785(52.6)			
Yes	15(1.0)	13(86.7)		13(86.7)		12(80)			
Cerebral infarction			<0.001		<0.001		<0.001		
No	1127(74.8)	548(48.6)		363(32.2)		530(47)			
Yes	379(25.2)	261(68.9)		229(60.4)		267(70.4)			
Cerebral atrophy			0.002		<0.001		<0.001		
No	925(61.4)	467(50.5)		293(31.7)		427(46.2)			
Yes	581(38.6)	342(58.9)		299(51.5)		370(63.7)			
Cerebral Hemorrhage			<0.001		<0.001		0.053		
No	1463(97.1)	774(52.9)		564(38.6)		768(52.5)			
Yes	43(2.9)	35(81.4)		28(65.1)		29(67.4)			
Brain trauma			0.664		0.639		0.542		
No	1375(91.3)	741(53.9)		538(39.1)		731(53.2)			
Yes	131(8.7)	68(51.9)		54(41.2)		66(50.4)			
Smoking			0.104		0.866		0.485		
No	1376(91.4)	748(54.4)		540(39.2)		732(53.2)			
Yes	130(8.6)	61(46.9)		52(40)		65(50)			
Drinking			<0.001		0.103		0.01		
No	1328(88.2)	740(55.7)		532(40.1)		719(54.1)			
Yes	178(11.8)	69(38.8)		60(33.7)		78(43.8)			
Depression			0.004		0.031		0.014		
No	1489(98.9)	794(53.3)		581(39)		783(52.6)			
Yes	17(1.1)	15(88.2)		11(64.7)		14(82.4)			
Anxiety			0.004		0.874		0.142		
No	1489(98.9)	794(53.3)		585(39.3)		785(52.7)			
Yes	17(1.1)	15(88.2)		7(41.2)		12(70.6)			
Insomnia			0.01		0.003		0.118		
No	1355(90.0)	713(52.6)		516(38.1)		708(52.3)			
Yes	151(10.0)	96(63.6)		76(50.3)		89(58.9)			

CDR, the clinical dementia rating scale. ADL, the activity of daily living scale. AD8, ascertain dementia 8. CSI-D, community screening instrument for dementia. Mini-cog, Mini-cognitive Assessment. VFT, the verbal fluency test. RAVLT, the rey auditory verbal learning test. CI, cognitive impairment.

### 3.2 Comparison of cognitive assessment between the seven assessments, and cognitive function by sociodemographic and health-related factors

**Figure 2:**
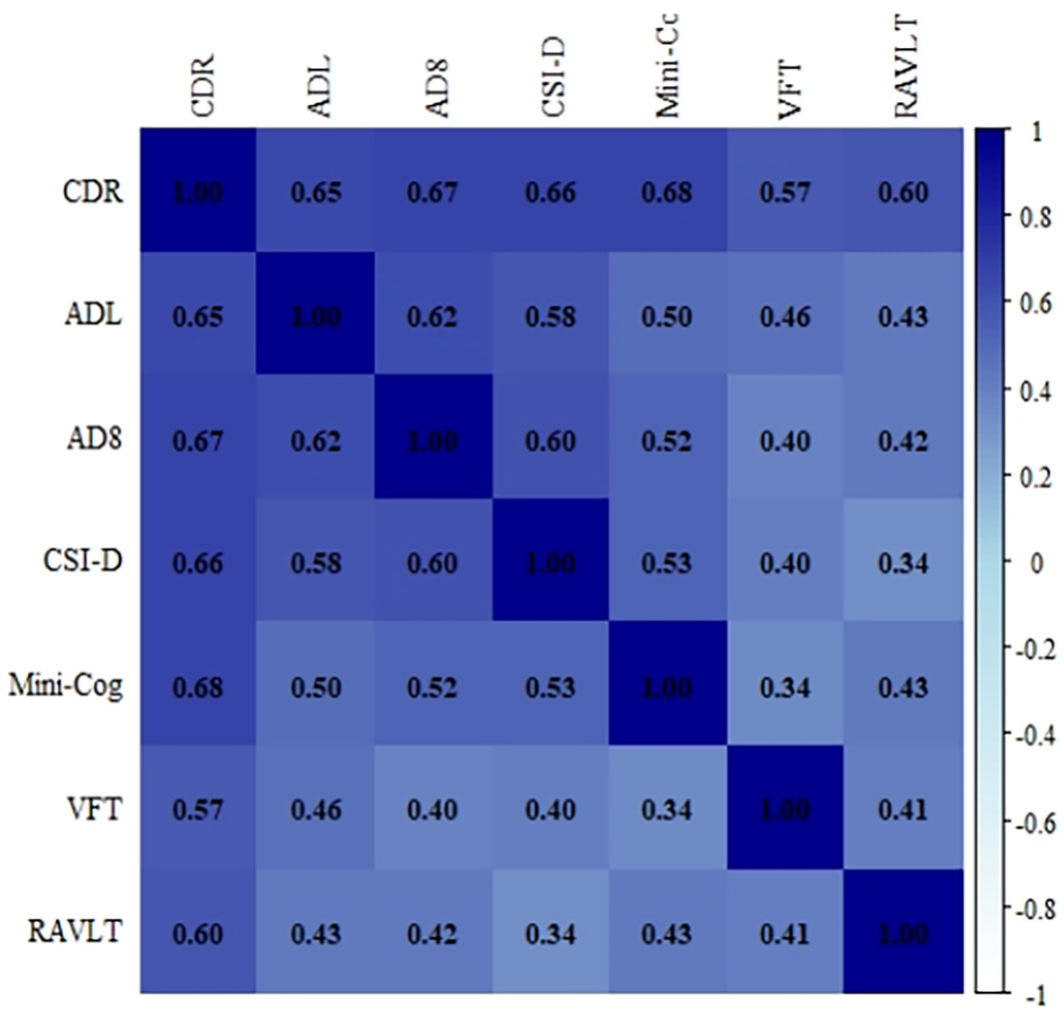
Inter-scale correlation coefficient matrix heat map (Cramer's V).

**Figure 3:**
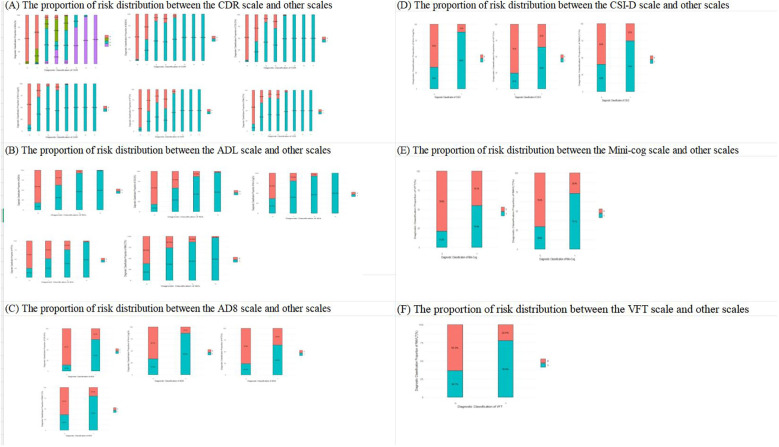
Risk distribution proportions of the seven scales.

According to the diagnostic criteria of the seven screening instruments (**Table 1**), the screening positivity proportions for CI identified by CDR, ADL, AD8, CSI-D, Mini-Cog, VFT, and RAVLT were 61.1%, 33.7%, 38.0%, 37.1%, 53.7%, 39.3%, and 52.9%, respectively (**Table 2**). The screening positivity proportions for CI rose significantly with advancing age group (*p* < 0.001); the ≥80-year group showed the highest proportions across instruments (CDR: 79.7%; ADL: 60.6%; AD8: 61.0%; CSI-D: 54.7%; Mini-Cog: 73.6%; VFT: 56.9%; RAVLT: 71.5%). In contrast, higher educational level was associated with lower screening positivity proportions for CI, producing a clear inverse trend (*p* < 0.001) (**Table 2**). Pairwise comparisons among the seven screening tools revealed positive correlations for every pair (Cramer’s V: 0.34–0.68, all *p* < 0.05; **Figure 2**). Concordance in CI classification across scales was also observed, indicating fair-to-moderate agreement(Kappa: 0.32–0.645, all *p* < 0.001; **Supplementary Table 2**). The risk distribution overlap for CI between any two scales exceeds 50%; that is, among subjects classified as positive by one scale, more than 50% are also classified as positive by any other scale (**Figure 3**).

### 3.3 Assessment of consistency among sociodemographic and health-related factors across the seven scales

Used a multivariable logistic regression to examine whether sociodemographic and health-related factors showed consistent associations across the seven scales. The analyses indicated strong consistency: age, education, hypertension, hyperglycemia, hyperlipidemia, coronary heart disease, cerebral infarction, and insomnia were all similarly associated with outcomes on the seven scales. In particular, participants aged ≥80 years had markedly higher odds of screening positivity for CI than the 60–69 year reference group across all scales (CDR: OR = 3.362, 95% CI: 2.4–4.71; ADL: OR = 6.177, 95% CI: 4.304–8.863; AD8: OR = 4.703, 95% CI: 3.357–6.589; CSI-D: OR = 3.078, 95% CI: 2.225–4.259; Mini-cog: OR = 3.766, 95% CI: 2.725–5.206; VFT: OR = 2.930, 95% CI: 2.126–4.037; RAVLT: OR = 2.939, 95% CI: 2.130–4.055) (**Table 3**). Compared with the illiterate reference group, participants with a primary school education had a significantly lower risk of screening positivity for CI across multiple assessments: CDR (OR = 0.491, 95% CI: 0.341–0.707), ADL (OR = 0.595, 95% CI: 0.416–0.850), AD8 (OR = 0.428, 95% CI: 0.306–0.597), CSI-D (OR = 0.586, 95% CI: 0.423–0.810), Mini-Cog (OR = 0.484, 95% CI: 0.344–0.681), VFT (OR = 0.652, 95% CI: 0.470–0.904), and RAVLT (OR = 0.670, 95% CI: 0.483–0.929) (**Table 3**). By contrast, a history of cerebral infarction was associated with a markedly higher screening positivity proportion for CI compared with those without infarction: CDR (OR = 1.994, 95% CI: 1.473–2.699), ADL (OR = 2.349, 95% CI: 1.76–3.135), AD8 (OR = 2.173, 95% CI: 1.646–2.868), CSI-D (OR = 2.090, 95% CI: 1.603–2.724), Mini-Cog (OR = 1.884, 95% CI: 1.424–2.494), VFT (OR = 2.473, 95% CI: 1.893–3.232), and RAVLT (OR = 2.555, 95% CI: 1.945–3.357) (both *p* < 0.05)(**Table 3**). In contrast, histories of hypertension, hyperglycemia, hyperlipidemia, coronary heart disease, and insomnia showed no statistically significant associations with screening positivity proportion for CI (*p*> 0.05) (**Table 3**).

**Table 3:** Multivariable logistic regression model assessing the associations of sociodemographic and health-related factors with the risk of screening-positive for CI across seven scales.

Variables	CI by ADL			CI by CDR			CI by AD8			CI by CSI-D		
	b	*p*-value	OR(95%CI)	b	*p*-value	OR(95%CI)	b	*p*-value	OR(95%CI)	b	*p*-value	OR(95%CI)
Gender(female)	-0.619	0.000	0.538(0.392,0.739)	-0.522	0.001	0.593(0.442,0.796)	-0.124	0.426	0.884(0.652,1.198)	-0.498	0.001	0.608(0.454,0.815)
Age(years)												
70-79	0.521	0.003	1.684(1.193,2.377)	0.182	0.193	1.199(0.912,1.578)	0.442	0.005	1.555(1.142,2.118)	0.464	0.003	1.59(1.177,2.149)
80-	1.821	0.000	6.177(4.304,8.863)	1.213	0.000	3.362(2.4,4.71)	1.548	0.000	4.703(3.357,6.589)	1.124	0.000	3.078(2.225,4.259)
Educational attainment												
primary school	-0.520	0.004	0.595(0.416,0.85)	-0.712	0.000	0.491(0.341,0.707)	-0.849	0.000	0.428(0.306,0.597)	-0.535	0.001	0.586(0.423,0.81)
junior high school or above	-0.309	0.079	0.734(0.52,1.036)	-1.350	0.000	0.259(0.181,0.371)	-0.858	0.000	0.424(0.306,0.588)	-0.749	0.000	0.473(0.344,0.649)
Marital status(yes)	-0.395	0.004	0.674(0.515,0.881)	-0.232	0.075	0.793(0.615,1.024)	-0.146	0.257	0.864(0.671,1.113)	-0.254	0.044	0.776(0.606,0.993)
Hypertension(yes)	0.009	0.947	1.009(0.778,1.308)	-0.015	0.905	0.986(0.777,1.25)	-0.051	0.679	0.95(0.747,1.21)	0.044	0.711	1.045(0.83,1.315)
Hyperglycemia(yes)	0.246	0.101	1.279(0.953,1.716)	0.131	0.352	1.14(0.865,1.504)						
Hyperlipidemia(yes)	-0.144	0.382	0.866(0.626,1.197)				0.043	0.777	1.044(0.775,1.406)			
Parkinson's Disease(yes)	0.580	0.030	1.786(1.057,3.017)	0.573	0.059	1.774(0.98,3.213)	0.457	0.076	1.58(0.953,2.618)			
Epilepsy(yes)	1.648	0.024	5.195(1.241,21.74)	1.682	0.118	5.378(0.652,44.388)	1.397	0.042	4.043(1.055,15.494)	1.539	0.026	4.66(1.203,18.044)
Coronary heart disease(yes)	-0.109	0.487	0.897(0.66,1.219)	-0.106	0.479	0.899(0.67,1.207)	-0.256	0.086	0.774(0.578,1.037)			
Cerebral Infarction(yes)	0.854	0.000	2.349(1.76,3.135)	0.690	0.000	1.994(1.473,2.699)	0.776	0.000	2.173(1.646,2.868)	0.737	0.000	2.09(1.603,2.724)
Cerebral Atrophy(yes)	0.345	0.009	1.412(1.091,1.827)	0.541	0.000	1.717(1.343,2.195)	0.498	0.000	1.645(1.293,2.093)	0.285	0.017	1.33(1.052,1.681)
Cerebral Hemorrhage(yes)	1.066	0.005	2.903(1.382,6.101)	0.761	0.085	2.141(0.899,5.097)	0.669	0.062	1.953(0.968,3.938)	0.695	0.044	2.004(1.02,3.941)
Brain trauma(yes)	-0.526	0.028	0.591(0.37,0.944)									
Smoking(yes)	-0.806	0.004	0.446(0.258,0.773)				-0.408	0.118	0.665(0.399,1.109)	-0.355	0.145	0.701(0.435,1.13)
Drinking(yes)	-0.368	0.123	0.692(0.434,1.104)	-0.511	0.007	0.6(0.414,0.87)	-0.365	0.101	0.694(0.449,1.074)	-0.294	0.164	0.745(0.492,1.127)
Depression(yes)	1.895	0.030	6.65(1.202,36.803)	20.183	0.998		0.447	0.463	1.563(0.474,5.149)	1.121	0.074	3.068(0.896,10.506)
Anxiety(yes)	1.124	0.059	3.078(0.957,9.901)				1.244	0.027	3.47(1.15,10.475)			
Insomnia(yes)	0.207	0.331	1.23(0.811,1.866)	0.401	0.059	1.493(0.984,2.265)	0.058	0.773	1.06(0.713,1.576)	0.190	0.326	1.21(0.827,1.769)
Constant	-0.921	0.001	0.398	1.104	0.000	3.016	-0.658	0.014	0.518	-0.383	0.142	0.682(0,0)
Variables	CI by Mini-cog			CI by VFT				CI by RAVLT				
	b	*p*-value	OR(95%CI)	b		*p*-value	OR(95%CI)	b		*p*-value		OR(95%CI)
Gender(female)	-0.411	0.004	0.663(0.5,0.879)	-0.518		0.000	0.596(0.457,0.777)	-0.550		0.000		0.577(0.435,0.764)
Age(years)												
70-79	0.240	0.085	1.272(0.968,1.671)	0.252		0.094	1.286(0.958,1.726)	0.251		0.096		1.285(0.957,1.727)
80-	1.326	0.000	3.766(2.725,5.206)	1.075		0.000	2.93(2.126,4.037)	1.078		0.000		2.939(2.13,4.055)
Educational attainment												
primary school	-0.725	0.000	0.484(0.344,0.681)	-0.428		0.010	0.652(0.47,0.904)	-0.401		0.016		0.67(0.483,0.929)
junior high school or above	-1.347	0.000	0.26(0.186,0.364)	-0.834		0.000	0.434(0.315,0.6)	-0.771		0.000		0.462(0.334,0.639)
Marital status(yes)	-0.024	0.846	0.976(0.762,1.249)	-0.049		0.703	0.952(0.741,1.224)	-0.050		0.694		0.951(0.74,1.222)
Hypertension(yes)				-0.005		0.968	0.995(0.79,1.254)	0.034		0.778		1.034(0.819,1.306)
Hyperglycemia(yes)	0.124	0.355	1.132(0.87,1.474)									
Hyperlipidemia(yes)								-0.234		0.116		0.792(0.592,1.059)
Parkinson's Disease(yes)	0.122	0.641	1.13(0.676,1.888)	0.277		0.274	1.319(0.804,2.165)	0.292		0.249		1.339(0.815,2.199)
Epilepsy(yes)	1.156	0.151	3.177(0.657,15.374)	1.912		0.017	6.765(1.413,32.392)	1.998		0.013		7.374(1.536,35.391)
Coronary heart disease(yes)	-0.139	0.329	0.87(0.658,1.15)									
Cerebral Infarction(yes)	0.634	0.000	1.884(1.424,2.494)	0.906		0.000	2.473(1.893,3.232)	0.938		0.000		2.555(1.945,3.357)
Cerebral Atrophy(yes)	0.116	0.337	1.123(0.887,1.421)	0.626		0.000	1.869(1.48,2.361)	0.646		0.000		1.908(1.51,2.41)
Cerebral Hemorrhage(yes)	1.049	0.012	2.855(1.264,6.446)	0.609		0.085	1.839(0.919,3.68)	0.622		0.080		1.863(0.929,3.737)
Brain trauma(yes)												
Smoking(yes)												
Drinking(yes)	-0.594	0.002	0.552(0.381,0.801)					-0.215		0.270		0.806(0.55,1.182)
Depression(yes)	1.125	0.167	3.081(0.626,15.174)	0.273		0.637	1.313(0.423,4.083)	0.324		0.574		1.382(0.447,4.274)
Anxiety(yes)	1.963	0.013	7.117(1.524,33.233)									
Insomnia(yes)	0.268	0.182	1.307(0.882,1.937)	0.240		0.220	1.271(0.866,1.865)					
Constant	0.681	0.008	1.975	-0.503		0.043	0.604	-0.457		0.075		0.633

The multivariate logistic regression model evaluated the relationship between sociodemographic and health-related factors and the risk of cognitive impairment (CI) under each scale. The reference groups used for comparison are as follows: aged between 60 and 69, male, illiterate, unmarried, without hypertension, hyperglycemia, hyperlipidemia, Parkinson's disease, epilepsy, coronary heart disease, cerebral infarction, brain atrophy, cerebral hemorrhage, brain trauma, non-smoking, non-drinking, no depression, no anxiety and no insomnia.

CDR, the clinical dementia rating scale. ADL, the activity of daily living scale. AD8, ascertain dementia 8. CSI-D, community screening instrument for dementia. Mini-cog, Mini-cognitive Assessment. VFT, the verbal fluency test. RAVLT, the rey auditory verbal learning test. CI, cognitive impairment.

### 3.4 Assessment of differences among sociodemographic and health-related factors across the seven scales

A multi-factor logistic regression model was employed to assess the differences in socio-demographic and health-related factors across the seven scales. The analysis revealed that factors such as gender, marital status, Parkinson’s disease, epilepsy, brain atrophy, cerebral hemorrhage, brain trauma, smoking, alcohol consumption, depression, and anxiety exhibited differing impacts on the seven scales. The specific findings are as follows: male subjects, unmarried individuals, and those with Parkinson’s disease, epilepsy, brain atrophy, cerebral hemorrhage, brain trauma, smoking, and depression showed an increased proportion of screening-positive CI on the ADL. In contrast, men, individuals with brain atrophy, and those who consume alcohol showed a heightened risk of screening-positive CI on the CDR. Furthermore, subjects with epilepsy, brain atrophy, and anxiety were at increased risk of screening-positive CI on the AD8. Male participants, those without a spouse, and individuals with epilepsy, brain atrophy, and cerebral hemorrhage also exhibited a higher risk of screening-positive CI on the CSI-D. Additionally, men, individuals with cerebral hemorrhage, alcohol consumption, and anxiety were found to have a greater risk of screening-positive CI on the Mini-Cog. Lastly, men with epilepsy and brain atrophy were associated with an increased risk of screening-positive CI on both the VFT and the RAVLT (all *p* < 0.05) (**Table 3**).

## 4 Discussion

This study found that, among people aged 60 and above in Chongqing, the screening positivity proportion for CI measured by CDR, ADL, AD8, CSI-D, Mini-Cog, VFT, and RAVLT was relatively high, Previous studies have also shown that the incidence of CI in Chongqing is higher than that in other cities in China (32), exceeding the reported national range (5.2-23.4%) (33) and surpassing rates observed in other countries (34, 35). Notably, the screening positivity proportion in Chongqing remained higher than that reported in the neighboring city of Chengdu (16.0%) (36). A major contributor to these disparities is likely heterogeneity in diagnostic criteria across studies, Prior work has used DSM-V, DSM-IV, MMSE, MoCA, Petersen/Winblad criteria, and other standards, producing different case definitions and prevalence estimates standards (35, 37, 38). That approach can inflate prevalence if a scale has a high false-positive rate. For example, CDR is primarily intended for early clinical detection of dementia; using it alone to screen for CI may produce an elevated false-positive rate and thus overestimate CI prevalence (39). This study focuses on how sociodemographic and health-related factors affect CI screening across seven scales and on differences among those scales; therefore, the CI prevalence reported here should be interpreted as a screening positivity proportion, suitable for comparing and analyzing the impact of factors across the seven instruments—but not as a clinical diagnosis or an epidemiological prevalence rate.

We selected sociodemographic and health-related variables for this study based on prior research and Chongqing’s local characteristics. For modifiable factors, we specifically considered conditions linked to local dietary habits, such as frequent consumption of hot pot; accordingly, we included indicators for hyperlipidemia, hyperglycemia, and hypertension. A comparison of the risk factors for the positive rate of CI screening in seven scales revealed that hyperlipidemia, hyperglycemia, and hypertension could increase the risk of positive CI screening, which is consistent with previous studies (24, 40). This association may reflect the effects of high-sugar and high-fat diets, which can promote oxidative stress and inflammatory responses in the brain and thereby cause neuronal damage and cognitive decline (41, 42). Second, modern stressors such as work pressure, a fast-paced lifestyle, and rapid urbanization contribute to anxiety, depression, and sleep disturbances. For this reason, we included insomnia, anxiety, and depression in the present study. Our results indicate that older adults with depression, anxiety, or insomnia are more likely to screen positive for CI, consistent with prior reports (43, 44). One plausible mechanism is that anxiety and depression produce abnormal changes in brain regions (for example, the prefrontal cortex and hippocampus) (45) and they dysregulate neurotransmitter and neuroendocrine systems, including serotonin and cortisol (46), thereby impairing attention, memory, and decision-making. In addition, several studies identify sleep as an important mediator of cognitive function in older adults: both short and long sleep durations can reduce cognition by weakening ADL and exacerbating depressive symptoms (47). Finally, the study found that epilepsy, cerebral infarction, and brain atrophy each significantly increased the risk of screening-positive for CI on the seven scales, a finding consistent with previous research (48). Because CI commonly results from intracranial pathology, abnormalities such as altered electroencephalogram signals, cerebrovascular disorders, or parenchymal brain lesions can all produce cognitive dysfunction.

In terms of unchangeable factors: This study found that the lower the educational level, the worse the results of the seven scales. Previous studies have also shown the relationship between CI and educational level, especially among groups with less than 6 years of education and illiterates, who have a higher risk of screening-positive CI (40, 49). This may be related to the fact that educational level is one of the indicators of cognitive reserve (50), that is, the higher the educational level, the more abundant the knowledge reserve of the elderly, and the higher the educational level, It is related to the fact that patients have more channels to acquire new knowledge, are more willing to abandon old ideas, accept new concepts and adjust their lifestyles in a timely manner. The relationship between age and screening positivity proportion for CI is contrary to that of culture: That is, the older the age, the higher the risk of screening-positive CI, especially for the elderly group aged 80 and above, the risk screening-positive of CI is the highest. This conclusion is consistent with previous research conclusions (40, 49) and may be related to factors such as the decline in the clearance ability of intracranial “toxic proteins”, namely Aβ and tau, lymphatic circulation obstruction, and nerve damage with the increase of age (26, 51). Considering that this study focuses on the positive rate of CI screening, not the prevalence of the disease, therefore, This conclusion indirectly confirms that CI shows a high degree of age dependence, and this factor is uncontrollable.

Notably, the distribution of sociodemographic and health-related factors affecting screening-positive for CI differs across the seven scales, but the key findings emerge. First, men show a higher risk of positive CI screening on six scales—ADL, CDR, CSI-D, Mini-cog, VFT, and RAVLT—whereas this result is not apparent on AD8. A likely explanation is that AD8 primarily screens for early Alzheimer’s disease and related dementias (ADRD), and not all CI cases progress to ADRD (8); thus AD8 has limited sensitivity for CI (MCI) (15). Second, although prevalence studies report higher CI rates in women, more severe cases tend to occur in men (52), which may account for the absence of clear early-stage dementia symptoms in men. In addition, women retain a lifelong advantage in verbal memory (53), and because verbal memory decline is among the earliest and most prominent cognitive deficits in AD patients, this advantage may contribute to sex differences in CI screening (54), Men are therefore less likely to be identified by the AD8. This finding also partly explains why men show a higher screening positivity proportion for CI on socially oriented functional scales such as the CDR, ADL, and CSI-D. Lastly, brain atrophy is associated with a higher risk of screening positivity CI on six scales (ADL, CDR, AD8, CSI-D, VFT, and RAVLT) but not on the Mini-Cog. A likely explanation is that many community-dwelling individuals with brain atrophy are asymptomatic or have only mild symptoms, so early memory deficits are subtle (55). In addition, the Mini-Cog combines a simple three-word recall test with a clock-drawing task, because the recall component is brief and low in difficulty (49), the Mini-Cog may be relatively insensitive to the mild memory impairment typical of early brain atrophy. Lastly, we note that the screening-positive rates on all seven scales were relatively high, with several scales classifying more than half of participants as positive. This raises concern about possible inflation of false positives. Therefore, we emphasize that these figures represent screening-positive rates from the scales, not true prevalence.

## 5 Limitations

This study has several limitations. First, we used seven scales and corresponding educational-attainment thresholds to screen for CI, which may have affected international comparisons of CI prevalence and its determinants. Second, limited data prevented inclusion of biochemical and neuroimaging markers to examine their associations with the seven scales, so we could not perform subclinical-symptom analyses of CI. Third, we did not use a diagnostic gold standard for CI, preventing comparison of the sensitivity and specificity of the seven scales. Future studies should apply established diagnostic criteria for CI (for example, the DSM-V standard), and collect biological markers whenever possible. Clinicians should then integrate medical history, changes in social and daily functioning, noncognitive behavioral symptoms, laboratory findings, and results from brain imaging and electrophysiology to reach a definitive diagnosis. Once patients are diagnosed and subtyped, researchers can analyze biological indicators and specific scale items to identify and explain subclinical CI symptoms. Finally, this study is cross-sectional, so identified risk factors pertain only to the positive rates obtained by screening scales and do not establish causal links with clinically diagnosed CD. Accordingly, our conclusions apply to screening performance rather than to CI diagnosis or population prevalence. Future work should compare each scale’s results with gold-standard diagnostic outcomes to quantify accuracy and specificity. To strengthen causal inference between scale positivity and risk factors, longitudinal follow-up with repeated assessments is needed.

## 6 Conclusion

Our results show that seven neuropsychological assessment scales correlate well and exhibit fair-to-moderate concordance in screening cognitive impairment (CI) among Chongqing residents aged 60 and older. All seven instruments produced a relatively high screening positivity proportion for CI in this sample. Beyond age, sex, and education, we identified several potentially modifiable risk factors associated with screening-positive CI, including marital status, epilepsy, cerebral infarction, brain atrophy, depression, anxiety, hyperglycemia, hyperlipidemia, and hypertension. Proactive management of these factors could reduce the proportion of positive CI screens. Furthermore, We also observed that different neuropsychological instruments are differentially influenced by sociodemographic and health-related characteristics. Therefore, community screening programs should select assessment tools that align with the target population’s characteristics and the local disease profile. In many community settings, minimizing missed diagnoses is the main priority and may justify accepting lower specificity. Accordingly, choosing scales that maximize sensitivity—and thus increase the positive detection rate—can help reduce false negatives. In summary, our findings indicate a high screening-positive CI among older adults in Chongqing. Careful selection of screening methods therefore provides a critical foundation for developing targeted interventions.

## Data Availability

The original contributions presented in the study are included in the article/**Supplementary Material**. Further inquiries can be directed to the corresponding authors.
