# Supplementary material for: A comparison between seven scales of neuropsychological assessments for cognitive impairment screening in Chinese older population: a cross-sectional study in Chongqing, China
Source: Front Psychiatry. 2026 Jul 23;17:1786091. doi: 10.3389/fpsyt.2026.1786091 (PMC13442869; doi:10.3389/fpsyt.2026.1786091)
Supplement: Supplementary file 1 [file DataSheet1.pdf]

Supplementary Material

A comparison between seven scales of neuropsychological assessments for cognitive impairment screening in Chinese older population: a cross-sectional study in Chongqing, China

Feiya Cai[1], Xiaolin Tan[*1], Bo Tian[*1]

[1]1. Department of Psychiatric Research, Chongqing Mental Health Center, Chongqing, China.

*** Correspondence:**

Bo Tian, Department of Psychiatric Research, Chongqing Mental Health Center, Chongqing 401147, China. Email: tianbo9460@163.com.

Xiaolin Tan, Department of Psychiatric Research, Chongqing Mental Health Center, Chongqing 401147, China. Email: tanxiaolincq@sina.com.

Keywords: ADL, agreement, CDR, correlation, cognitive impairment, Mini-Cog, risk factors.

Table S1 Multicollinearity detection for seven scales

	collinearity statistics (CDR)			collinearity statistics (ADL)			collinearity statistics (AD8)			collinearity statistics(CSI-D)	
	Tolerance	VIF		Tolerance	VIF		Tolerance	VIF		Tolerance	VIF
Gender	0.714537576	1.399506526	Gender	0.800670161	1.24895375	Gender	0.715311921	1.397991521	Gender	0.722974256	1.383175116
Age	0.814864276	1.227198233	Age	0.819033411	1.220951412	Age	0.815571997	1.226133319	Age	0.834993821	1.197613653
Educational attainment	0.921254096	1.085476857	Educational attainment	0.942132308	1.061422044	Educational attainment	0.921974608	1.08462857	Educational attainment	0.942698398	1.060784661
Marital status	0.827820469	1.20799139	Marital status	0.830543929	1.204030232	Marital status	0.827845446	1.207954945	Marital status	0.83207744	1.201811216
Hypertension	0.891481421	1.121728368	Hypertension	0.899628471	1.111569978	Hypertension	0.912516149	1.095871016	Hypertension	0.947367264	1.055556844
Hyperglycemia	0.915385363	1.092436082	Hyperglycemia	0.951610347	1.050850281	Hyperlipidemia	0.861480147	1.160792855			
Hyperlipidemia	0.827396954	1.208609719									
Parkinson's Disease	0.959700838	1.041991379	Parkinson's Disease	0.96405876	1.037281172	Parkinson's Disease	0.961281351	1.040278165			
Epilepsy	0.963239874	1.038163003	Epilepsy	0.966656993	1.034493111	Epilepsy	0.964327006	1.036992632	Epilepsy	0.968944908	1.032050421
Coronary heart disease	0.883915608	1.131329723	Coronary heart disease	0.905420301	1.104459441	Coronary heart disease	0.884764601	1.130244134			
Cerebral Infarction	0.837725722	1.193708124	Cerebral Infarction	0.852209256	1.173420722	Cerebral Infarction	0.839247838	1.191543135	Cerebral Infarction	0.867293006	1.153012872
Cerebral Atrophy	0.926176908	1.079707334	Cerebral Atrophy	0.931355908	1.073703395	Cerebral Atrophy	0.92981944	1.075477622	Cerebral Atrophy	0.941548608	1.062080058
Cerebral Hemorrhage	0.960986238	1.040597628	Cerebral Hemorrhage	0.972130029	1.028668974	Cerebral Hemorrhage	0.970218381	1.03069579	Cerebral Hemorrhage	0.971502402	1.029333533
Brain trauma	0.971255922	1.029594752									
Smoking	0.732192932	1.365760248				Smoking	0.73224608	1.365661118	Smoking	0.733551708	1.363230415

Drinking	0.780189598	1.281739724	Drinking	0.868163032	1.151857385	Drinking	0.781392512	1.27976655	Drinking	0.781615413	1.279401587
Depression	0.935860219	1.068535642	Depression	0.957926593	1.043921327	Depression	0.937193834	1.067015129	Depression	0.958815148	1.042953902
Anxiety	0.945317804	1.057845305				Anxiety	0.947240354	1.055698267			
Insomnia	0.913368695	1.094848122	Insomnia	0.938133788	1.065946044	Insomnia	0.91378381	1.094350752	Insomnia	0.939229276	1.064702757

Table S1 (Continued)

	**collinearity statistics (Mini-cog)**			**collinearity statistics (VFT)**			**collinearity statistics (RAVLT)**	
	**Tolerance**	**VIF**		**Tolerance**	**VIF**		**Tolerance**	**VIF**
Gender	0.806577354	1.239806691	Gender	0.905124863	1.104819943	Gender	0.808918675	1.236218215
Age	0.820348505	1.218994116	Age	0.846047705	1.181966447	Age	0.841943791	1.187727745
Educational attainment	0.943058141	1.060380009	Educational attainment	0.943491278	1.059893211	Educational attainment	0.935477698	1.068972571
Marital status	0.833453262	1.199827328	Marital status	0.832674467	1.200949518	Marital status	0.831486636	1.20266515
			Hypertension	0.947412724	1.055506196	Hypertension	0.929574095	1.075761475
Hyperglycemia	0.98051265	1.019874654						
						Hyperlipidemia	0.893921534	1.118666417
Parkinson's Disease	0.960358415	1.030278165	Parkinson's Disease	0.940335845	1.020278165	Parkinson's Disease	0.930335845	1.040278165
Epilepsy	0.968781737	1.032224249	Epilepsy	0.969408597	1.031556769	Epilepsy	0.972912853	1.027841288
Coronary heart disease	0.926001785	1.079911526						
Cerebral Infarction	0.8686979	1.151148172	Cerebral Infarction	0.873283759	1.145103169	Cerebral Infarction	0.849061529	1.177770945
Cerebral Atrophy	0.936924685	1.067321649	Cerebral Atrophy	0.942067881	1.061494634	Cerebral Atrophy	0.943482468	1.059903108
Cerebral Hemorrhage	0.972355411	1.02843054	Cerebral Hemorrhage	0.973243556	1.027492033	Cerebral Hemorrhage	0.972156336	1.028641138
Drinking	0.868083029	1.151963541				Drinking	0.868758129	1.151068367

Depression	0.938029779	1.066064236	Depression	0.959259883	1.042470364	Depression	0.964597155	1.036702207
Anxiety	0.948090654	1.054751458						
Insomnia	0.925643772	1.080329204	Insomnia	0.939560991	1.064326861			

Abbreviations: CDR, the clinical dementia rating scale. ADL, the activity of daily living scale. AD8, ascertain dementia 8. CSI-D, community screening instrument for dementia. Mini-cog, Mini-cognitive Assessment. VFT, the verbal fluency test. RAVLT, the rey auditory verbal learning test.

Table S2 Consistency testing of the seven scales for CI classification

		CDR					ADL					AD8					CSI-D				
		CI,n(%)	no CI,n(%)	Total,n(%)	Kappa	*p*-value	CI,n(%)	no CI,n(%)	Total,n(%)	Kappa	*p*-value	CI,n(%)	no CI,n(%)	Total,n(%)	Kappa	*p*-value	CI,n(%)	no CI,n(%)	Total,n(%)	Kappa	*p*-value
**CDR**	CI,n(%)						480(31.9)	440(29.2)	920(61.1)	0.422	<0.001	556(36.9)	364(24.2)	920(61.1)	0.519	<0.001	537(35.7)	383(25.4)	920(61.1)	0.491	<0.001
	no CI,n(%)						27(1.8)	559(37.1)	586(38.9)			17(1.1)	569(37.8)	586(38.9)			22(1.5)	564(37.5)	586(38.9)		
	Total,n(%)						507(33.7)	999(66.3)	1506(100)			573(38)	933(62)	1506(100)			559(37.1)	947(62.9)	1506(100)		
**ADL**	CI,n(%)	480(31.9)	27(1.8)	507(33.7)								397(26.4)	110(7.3)	507(33.7)	0.588	<0.001	377(25)	130(8.6)	507(33.7)	0.548	<0.001
	no CI,n(%)	440(29.2)	559(37.1)	999(66.3)								176(11.7)	823(54.6)	999(66.3)			182(12.1)	817(54.2)	999(66.3)		
	Total,n(%)	920(61.1)	586(38.9)	1506(100)								573(38)	933(62)	1506(100)			559(37.1)	947(62.9)	1506(100)		
**AD8**	CI,n(%)	556(36.9)	17(1.1)	573(38)			397(26.4)	176(11.7)	573(38)								426(28.3)	147(9.8)	573(38)	0.604	<0.001
	no CI,n(%)	364(24.2)	569(37.8)	933(62)			110(7.3)	823(54.6)	933(62)								133(8.8)	800(53.1)	933(62)		
	Total,n(%)	920(61.1)	586(38.9)	1506(100)			507(33.7)	999(66.3)	1506(100)								559(37.1)	947(62.9)	1506(100)		

		CDR			ADL			AD8			CSI-D		
**CSI-D**	CI,n(%)	537(35.7)	22(1.5)	559(37.1)	377(25)	182(12.1)	559(37.1)	426(28.3)	133(8.8)	559(37.1)			
	no CI,n(%)	383(25.4)	564(37.5)	947(62.9)	130(8.6)	817(54.2)	947(62.9)	147(9.8)	800(53.1)	947(62.9)			
	Total,n(%)	920(61.1)	586(38.9)	1506(100)	507(33.7)	999(66.3)	1506(100)	573(38)	933(62)	1506(100)			
**Mini-cog**	CI,n(%)	733(48.7)	76(5)	809(53.7)	445(29.5)	364(24.2)	809(53.7)	498(33.1)	311(20.7)	809(53.7)	492(32.7)	317(21)	809(53.7)
	no CI,n(%)	187(12.4)	510(33.9)	697(46.3)	62(4.1)	635(42.2)	697(46.3)	75(5)	622(41.3)	697(46.3)	67(4.4)	630(41.8)	697(46.3)
	Total,n(%)	920(61.1)	586(38.9)	1506(100)	507(33.7)	999(66.3)	1506(100)	573(38)	933(62)	1506(100)	559(37.1)	947(62.9)	1506(100)
**VFT**	CI,n(%)	539(35.8)	53(3.5)	592(39.3)	341(22.6)	251(16.7)	592(39.3)	367(24.4)	255(16.9)	622(41.3)	362(24)	230(15.3)	592(39.3)
	no CI,n(%)	381(25.3)	533(35.4)	914(60.7)	166(11)	748(49.7)	914(60.7)	176(11.7)	708(47)	884(58.7)	197(13.1)	717(47.6)	914(60.7)
	Total,n(%)	920(61.1)	586(38.9)	1506(100)	507(33.7)	999(66.3)	1506(100)	543(36.1)	963(63.9)	1506(100)	559(37.1)	947(62.9)	1506(100)
**RAVLT**	CI,n(%)	697(46.3)	100(6.6)	797(52.9)	417(27.7)	380(25.2)	797(52.9)	458(30.4)	339(22.5)	797(52.9)	418(27.8)	379(25.2)	797(52.9)
	no CI,n(%)	223(14.8)	486(32.3)	709(47.1)	90(6)	619(41.1)	709(47.1)	115(7.6)	594(39.4)	709(47.1)	141(9.4)	568(37.7)	709(47.1)
	Total,n(%)	920(61.1)	586(38.9)	1506(100)	507(33.7)	999(66.3)	1506(100)	573(38)	933(62)	1506(100)	559(37.1)	947(62.9)	1506(100)

Table S2 (Continued)

		**Mini-cog**					**VFT**					**RAVLT**				
		CI,n(%)	no CI,n(%)	Total,n(%)	Kappa	*p*-value	CI,n(%)	no CI,n(%)	Total,n(%)	Kappa	*p*-value	CI,n(%)	no CI,n(%)	Total,n(%)	Kappa	*p*-value
**CDR**	CI,n(%)	733(48.7)	187(12.4)	920(61.1)	0.645	<0.001	539(35.8)	381(25.3)	920(61.1)	0.45	<0.001	697(46.3)	223(14.8)	920(61.1)	0.565	<0.001

		Mini-cog					VFT					RAVLT				
	no CI,n(%)	76(5)	510(33.9)	586(38.9)			53(3.5)	533(35.4)	586(38.9)			100(6.6)	486(32.3)	586(38.9)		
	Total,n(%)	809(53.7)	697(46.3)	1506(100)			592(39.3)	914(60.7)	1506(100)			797(52.9)	709(47.1)	1506(100)		
**ADL**	CI,n(%)	445(29.5)	62(4.1)	507(33.7)	0.448	<0.001	341(22.6)	166(11)	507(33.7)	0.405	<0.001	417(27.7)	90(6)	507(33.7)	0.388	<0.001
	no CI,n(%)	364(24.2)	635(42.2)	999(66.3)			251(16.7)	748(49.7)	999(66.3)			380(25.2)	619(41.1)	999(66.3)		
	Total,n(%)	809(53.7)	697(46.3)	1506(100)			592(39.3)	914(60.7)	1506(100)			797(52.9)	709(47.1)	1506(100)		
**AD8**	CI,n(%)	498(33.1)	75(5)	573(38)	0.496	<0.001	367(24.4)	176(11.7)	543(36.1)	0.397	<0.001	458(30.4)	115(7.6)	573(38)	0.405	<0.001
	no CI,n(%)	311(20.7)	622(41.3)	933(62)			255(16.9)	708(47)	963(63.9)			339(22.5)	594(39.4)	933(62)		
	Total,n(%)	809(53.7)	697(46.3)	1506(100)			622(41.3)	884(58.7)	1506(100)			797(52.9)	709(47.1)	1506(100)		
**CSI-D**	CI,n(%)	492(32.7)	67(4.4)	559(37.1)	0.5	<0.001	362(24)	197(13.1)	559(37.1)	0.4	<0.001	418(27.8)	141(9.4)	559(37.1)	0.320	<0.001
	no CI,n(%)	317(21)	630(41.8)	947(62.9)			230(15.3)	717(47.6)	947(62.9)			379(25.2)	568(37.7)	947(62.9)		
	Total,n(%)	809(53.7)	697(46.3)	1506(100)			592(39.3)	914(60.7)	1506(100)			797(52.9)	709(47.1)	1506(100)		
**Mini-cog**	CI,n(%)						444(29.5)	365(24.2)	809(53.7)	0.329	<0.001	591(39.2)	218(14.5)	809(53.7)	0.434	<0.001
	no CI,n(%)						148(9.8)	549(36.5)	697(46.3)			206(13.7)	491(32.6)	697(46.3)		
	Total,n(%)						592(39.3)	914(60.7)	1506(100)			797(52.9)	709(47.1)	1506(100)		
**VFT**	CI,n(%)	444(29.5)	148(9.8)	592(39.3)								462(30.7)	130(8.6)	592(39.3)	0.39	<0.001
	no CI,n(%)	365(24.2)	549(36.5)	914(60.7)								335(22.2)	579(38.4)	914(60.7)		
	Total,n(%)	809(53.7)	697(46.3)	1506(100)								797(52.9)	709(47.1)	1506(100)		
**RAVLT**	CI,n(%)	591(39.2)	206(13.7)	797(52.9)			462(30.7)	335(22.2)	797(52.9)							

	Mini-cog			VFT			RAVLT
no CI,n(%)	218(14.5)	491(32.6)	709(47.1)	130(8.6)	579(38.4)	709(47.1)	
Total,n(%)	809(53.7)	697(46.3)	1506(100)	592(39.3)	914(60.7)	1506(100)	

Abbreviations: CDR, the clinical dementia rating scale. ADL, the activity of daily living scale. AD8, ascertain dementia 8. CSI-D, community screening instrument for dementia. Mini-cog, Mini-cognitive Assessment. VFT, the verbal fluency test. RAVLT, the rey auditory verbal learning test. CI: cognitive impairment.
